# Assessment of a program for monitoring antimicrobial purchase and resistance in *Escherichia coli* and *Salmonella enterica* on pig farms in the Midwestern United States from May 2020 through October 2023

**DOI:** 10.3389/fvets.2025.1586008

**Published:** 2025-07-25

**Authors:** Karyn A. Havas, Roy Edler, Laura Ruesch, Marlee Braun, Peter Ferm, Noelle R. Noyes, Laura B. Goodman, H. Morgan Scott, Joel Nerem, Taylor Spronk, Scott A. Dee

**Affiliations:** ^1^Pipestone Research, Pipestone, MN, United States; ^2^Animal Disease Research and Diagnostic Laboratory, South Dakota State University, Brookings, SD, United States; ^3^Department of Veterinary Population Medicine, College of Veterinary Medicine, University of Minnesota, St. Paul, MN, United States; ^4^Department of Public and Ecosystem Health, College of Veterinary Medicine, Cornell University, Ithaca, NY, United States; ^5^Department of Veterinary Pathobiology, College of Veterinary Medicine and Biomedical Sciences, Texas A&M University, College Station, TX, United States; ^6^Pipestone Veterinary Services, Pipestone, MN, United States

**Keywords:** swine, pigs, antimicrobial use, antimicrobial resistance, *Escherichia coli*, Salmonella

## Abstract

**Introduction:**

Antimicrobial resistance (AMR) poses significant challenges to health and treatment options in both human and veterinary medicine. Animal AMR monitoring in the US evaluates carcasses, retail meat, live animals, and diagnostic laboratory submissions; however, there is a lack of consistent on-farm monitoring of use and resistance.

**Methods:**

In 2020, 153 pig farms in the Midwestern United States enrolled in an antimicrobial purchase and resistance monitoring program. Intestinal samples or fecal swabs were collected biannually for 3 years from pigs and their dunging areas; antibiotic purchase data were tracked. *Salmonella enterica* and *Escherichia coli* were isolated and underwent antibiotic susceptibility testing using either a commercial bovine/ porcine (BOPO 7F) panel (for pig samples) or the National Antimicrobial Resistance Monitoring System (NARMS) Gram-negative panel (for dunging area samples). Minimum inhibitory concentrations (MICs) of antibiotics were used to evaluate the susceptibility of pig sample isolates, while NARMS breakpoints were used to assess resistance in isolates from dunging areas.

**Results:**

Tetracyclines were the most purchased, and penicillins were the most used antibiotic class across farm types. For pig samples, more isolates exhibited MIC values at the high end of the tested range among *E. coli* and *Salmonella* isolates from wean-to-market (WTM) sites compared to breed-to-wean (BTW) sites for almost all antibiotic classes. In addition, *E. coli* isolates from sick pigs had higher MIC values compared to isolates from substandard but otherwise healthy pigs. Among the dunging area isolates, both bacteria had higher rates of resistance in the WTM sites compared to the BTW sites across multiple antibiotics.

**Discussion:**

Individual ages of pigs were a likely confounder and were not controlled for, as these data were not reliably collected. A greater frequency of monitoring, along with controlling for age, recent treatments, and disease events at the individual level, would improve farm-level insights from on-farm AMR monitoring. Currently, the interpretation of phenotypic AMR data for resistance monitoring in swine medicine is limited by the lack of established veterinary breakpoints for enteric organisms. The available NARMS breakpoints are designed for humans, can be used for public health monitoring, and are likely to be applicable primarily to gastrointestinal infections involving the same bacteria in farm animals.

## 1 Introduction

Antimicrobial resistance (AMR) is a concern for human, plant, and animal health as it limits the effectiveness of antibiotics used to treat pathogenic bacteria. Recent studies highlighted the burden of AMR-related diseases on human health. Research published in 2019 provided estimated age-standardized human mortality rates in the United States for deaths due to AMR compared to never having been infected; for this endpoint, there were 30.7 deaths per 100,000 and 7.4 deaths per 100,000, respectively. *Escherichia coli* was one of the top contributing pathogens to AMR deaths (1). Other studies have shown that *E. coli*, which is resistant to third-generation cephalosporins and fluoroquinolones, as well as carbapenem-resistant *Klebsiella pneumoniae,* are responsible for 50,000 to 100,000 deaths each year (2). For animal health impacts, the American Veterinary Medical Association released a report titled “Antimicrobial Resistant Pathogens Affecting Animal Health in the United States” in 2020. This report summarized pathogens of concern with detected resistance. For pigs, these pathogens included *Salmonella enterica* and *E. coli,* among others (3). Despite the burden of disease in both humans and livestock, there is no scientific consensus that *E. coli* isolates with AMR causing severe disease in humans are related to livestock isolates (4). This is because these human cases are of septicemia or bloodstream infections. Nonetheless, there is well-documented direct contact, environmental, and foodborne transmission of pathogens between humans and livestock (5, 6), and this relationship should be monitored and further studied. Monitoring does not require the use of pathogenic strains alone. Commensal *E. coli* is recognized as an indicator organism for the AMR patterns in the broader *Enterobacteriaceae* population (7).

Several factors contribute to the selection pressure for antimicrobial resistance (AMR) in pathogenic bacteria, including the use of antimicrobials. The first U. S. National Action Plan for Combating Antibiotic-Resistant Bacteria (CARB) in 2015 recognized the need for enhanced monitoring of AMR patterns, as well as antimicrobial sales, use (AMU), and management practices in livestock production. The second CARB National Action Plan, created with input from over 20 federal agencies across all sectors, reiterated this need (8). The Food and Drug Administration (FDA) has regulatory oversight of antibiotic use in livestock production. In 2012, the FDA’s Center for Veterinary Medicine (CVM) set in motion actions to limit livestock use of medically important antibiotics (defined as antimicrobial agents important for therapeutic use in humans) to labels for treatment, control, or prevention of disease and thus ceased to allow the use of these antibiotics for growth promotion or improved feed efficiency (9). CVM also required drug sponsors to remove any growth-promoting language from packaging for water- and feed-administered drugs and further required veterinary oversight; the transition to these revised labels occurred on January 1, 2017. Effective June 2023, a veterinary prescription and a veterinarian-client-patient relationship (VCPR) became required for any of the few remaining over-the-counter food animal antibiotics considered medically important (10). These regulations are centered around human health needs. To understand the current use of antimicrobials in livestock, it is also essential to comprehend veterinary treatment needs and challenges, facilitating improved planning and a One Health approach.

The United States has several nationwide AMR monitoring programs involving animal products and populations. In 1996, the National Antimicrobial Resistance Monitoring System (NARMS) was established. It is a partnership among the Centers for Disease Control and Prevention (CDC), the FDA of the Department of Health and Human Services (DHHS), and the Food Safety and Inspection Service (FSIS) of the Department of Agriculture (USDA). NARMS monitors AMR in select enteric bacteria from human clinical cases (CDC), animal cecal contents, and meat products at slaughterhouses (FSIS), as well as in retail meats (FDA) (11). Furthermore, the Animal and Plant Health Inspection Service’s (APHIS) National Animal Health Laboratory Network (NAHLN) initiated the NAHLN AMR pilot project in 2018, with participation from 31 different laboratories as of 2022. This latter project monitors data from four livestock and two companion animal species. Similarly, the FDA’s Veterinary Laboratory Investigation and Response Network (Vet-LIRN) has conducted antibiotic susceptibility testing on animal isolates from diagnostic animal cases since 2017 (12, 13). Vet-LIRN also conducts whole-genome sequencing on a subset of isolates. The USDA’s National Animal Health Monitoring System (NAHMS) has collected data about AMU, stewardship, and AMR in its regular surveys since 1992 and has included these data in 17 different studies to date^1^. Most recently, NAHMS included a collection of AMU and stewardship data in its 2021 pig study for large enterprises (14). These efforts provide publicly available information on resistance patterns and MIC distributions for bacterial isolates from pig populations. There are a handful of publications regarding pathogens of pigs based on laboratory data rather than a longitudinal sampling of farm sites (15, 16); however, there remains a pressing need for on-farm monitoring of both sick and healthy animals to provide additional insights regarding on-farm patterns of bacterial resistance that occur where animals are raised and how those are related to AMU.

According to the 2022 US Census of Agriculture, there are 60,809 hog farmers in the United States, with a total hog inventory of 73,817,751 hogs. The majority of farms have ≤ 24 hogs (43,490), but the majority of hogs (55,528,543) are found on farms with more than 5,000 pigs on site (17). These larger facilities likely represent commercial farming in the United States. In the large farm category, there were 2,173 farms, and 542 (25.4%) were breed-to-wean, 705 (33.0%) were breed-to-finish, 1,641 (76.8%) were finish-only, 54 (2.5%) were breed-to-feeder, 331 (15.5%) were nurseries, and 267 (12.5%) were other types (17). The 2021 National Animal Health Monitoring Service study on large-scale swine enterprises (enterprises with ≥ 1,000 pigs) further describes this industry sector. Most enterprises in this sector (47%) had ≥ 5,000 hogs. Of the sites with ≥ 5,000 hogs, 33.2% of their inventory consisted of sows and unmated young females (gilts) that would be bred, 32.9% were nursing pigs, 65.4% were < 60-pound weaned hogs, and 84.8% had ≥ 60-pound market hogs. In this study, among the ≥ 5,000 head farms, 12.3% were breeding sites. A total of 41.8% were nursery-age pigs, and 73.5% were grower-finisher sites (18). The numbers do not tally to 100%, as sites with sows also have nursing pigs, and producers may own < 60-pound and ≥ 60-pound pigs as well as all pig types, depending upon the operation set-up.

This paper summarizes results from a monitoring program that included: (1) testing the antibiotic susceptibility of *S. enterica* and *E. coli* isolates from samples collected on pig farms from 2020 to 2023 and (2) antibiotic purchase records by drug class across commercial pig production systems. The strengths and weaknesses of the on-farm approach and the feasibility of maintaining the program over time are considered.

## 2 Materials and methods

### 2.1 Study design and farm enrollment

This program was based on a longitudinal sampling scheme that relied on convenience sampling of pigs and their environments from over 150 farm sites. The effort was to represent results from pig farms within the upper Midwest of the United States, which included a large swine production system (mostly breed-to-wean) as well as individual family farms (some breed-to-wean and all wean-to-market) throughout the region. Monitoring was initially planned for three time periods: May 2020 through May 2021, June 2021 through June 2022, and July 2022 through July 2023; however, sampling continued through October 2023. Enrolled farm enterprises had antibiotic purchases tracked using a centralized system called the Pipestone Antibiotic Resistance Tracker (PART). The farm sites were in Iowa, Illinois, Indiana, Minnesota, Missouri, North Dakota, South Dakota, and Wisconsin. To maintain client privacy, further breakdowns of farms and sites are not provided herein. Farm enterprises were categorized into three production models: breed-to-wean (BTW), wean-to-market (WTM), and breed-to-market (BTM). BTW, enterprise sites had female pigs that were bred and gave birth to piglets. Weaned piglets were then sold to WTM Enterprises to grow until they were harvested. BTM Farm enterprises had both BTW and WTM sites, but only WTM sites were sampled. The antibiotic susceptibility testing for isolates from the AST for BTM enterprises was thus described as WTM sites in this study. The sampling was planned to be completed twice in each period or approximately every 6 months. Many WTM and BTM enterprises had multiple sites as part of their farm enterprise, and this resulted in two different sites being sampled in any given period. The purpose was to capture *E. coli* and *Salmonella* isolates from pig samples at these farm sites.

### 2.2 Sampling

This program was planned as a descriptive study, and since *E. coli* is a commensal bacterium, sampling numbers were established based on producer and veterinary constraints. During periods one and two, samples were collected from two clinically ill and two substandard pigs twice per year. Substandard pigs were smaller than their peers, were not visibly ill, and had not received antibiotics. Substandard pigs were selected, as farmers were unwilling to sacrifice healthy pigs for the study. This allowed for an understanding of the impact of health on pathogen antibiotic susceptibility. Pig selection for sampling was at the veterinarian’s discretion. Substandard pig samples were pooled, and sick pig samples were pooled due to financial constraints. For each substandard and sick pig, the cecum and small intestine were collected as one sample. During period three, rectal fecal swabs were taken from four healthy pigs using BBL™ culture swabs (Becton, Dickinson, and Company, Franklin Lakes, New Jersey) and then were pooled. The time since the last antibiotic treatment for the sick pigs was not recorded. The change was made to address producers’ concerns about sampling time demands and to make the sampling more affordable and efficient. All the selected pigs were humanely euthanized following AVMA euthanasia guidelines (19). Additionally, in all periods, a composite fecal sample was collected from dunging areas using an environmental sampling sponge stick (3 M, Saint Paul, Minnesota). In the BTW sites, the following sites were swabbed using one swab: the dunging areas at the back of 25 farrowing crates in the oldest piglet room and at the back of 50 gestation crates from sows that just weaned their piglets, the weaned piglet holding pen and loading ramp to the truck and trailer, and five pens that held the oldest cohort of the female pigs that had not yet been bred (gilts). At the WTM sites, the dunging areas of the five pens housing the oldest pigs were swabbed to better represent the isolates present in pigs immediately prior to harvest. This project was approved by the Pipestone Institutional Care and Use Committee (protocol number 2020–001).

### 2.3 Bacterial isolation, characterization, and antimicrobial susceptibility testing

Pooled and composite fecal samples were sent to the South Dakota State University’s (SDSU) Animal Disease Research and Diagnostic Laboratory (ADRDL). Tissue and fecal swab samples were processed using standard ADRDL methodologies. The culture was performed using tryptic soy agar with sheep’s blood (blood agar) and tergitol-7 agar for the isolation of *E. coli* (20–22). If multiple colony morphologies of *E. coli* were observed, all morphologies were isolated and tested; otherwise, a representative isolate was identified and selected for further study. The *Salmonella* culture method is modified from the USDA Food and Safety Inspection Services (FSIS) Microbiology Laboratory Guidebook (23) and the National Poultry Improvement Plan Program Standard B (24). Buffered peptone water (BPW) pre-enrichment broth was inoculated with samples at a 1:10 dilution and incubated at 37°C ± 2°C without carbon dioxide (CO_2_) for 20 to 24 h. Tetrathionate and Rappaport-Vassiliadis enrichment broths were inoculated with incubated BPW pre-enrichment broth at a 1:10 and 1:100 dilution, respectively, and incubated at 42°C ± 2°C without CO_2_ for 20 to 24 h. Enrichment broths were plated onto XLT-4 and brilliant green with novobiocin agar with a 10 μL loop and incubated at 37°C ± 2°C with CO_2_ for 24 and 48 h, respectively. If multiple serogroups of *S. enterica* were isolated, all serogroups were tested. Composite fecal samples were cultured using the FDA NARMS method for retail meat surveillance (25) to isolate *E. coli* and *S. enterica*. Suspect representative isolates had their genus and species confirmed using matrix-assisted laser desorption/ionization-time of flight (MALDI-TOF) technology via the MALDI Biotyper® (Bruker, Billerica, Massachusetts).

Isolates were then tested to determine their antibiotic minimum inhibitory concentration (MIC) via broth microdilution using the Sensititre system (Thermo Fisher Scientific, Waltham, Massachusetts), with results read by the SWIN software system (Thermo Fisher Scientific, Waltham, Massachusetts). Isolates from tissues and fecal swabs were tested for antibiotic susceptibility using Bovine/Porcine 7F (BOPO7F) veterinary plates (Thermo Fisher Scientific, Waltham, Massachusetts), while composite fecal sample isolates were tested using the NARMS antimicrobial gram-negative plates. The project started with the NARMS CMV3AGNF Gram-negative plate (Thermo Fisher Scientific, Waltham, Massachusetts) but transitioned to the NARMS CMV5AGNF Gram-negative plate (Thermo Fisher Scientific, Waltham, Massachusetts) in October 2021. The latter plate changed the range of azithromycin testing, added meropenem and colistin, and removed streptomycin and ceftiofur.

### 2.4 Whole genome sequencing

**Figure 1:**
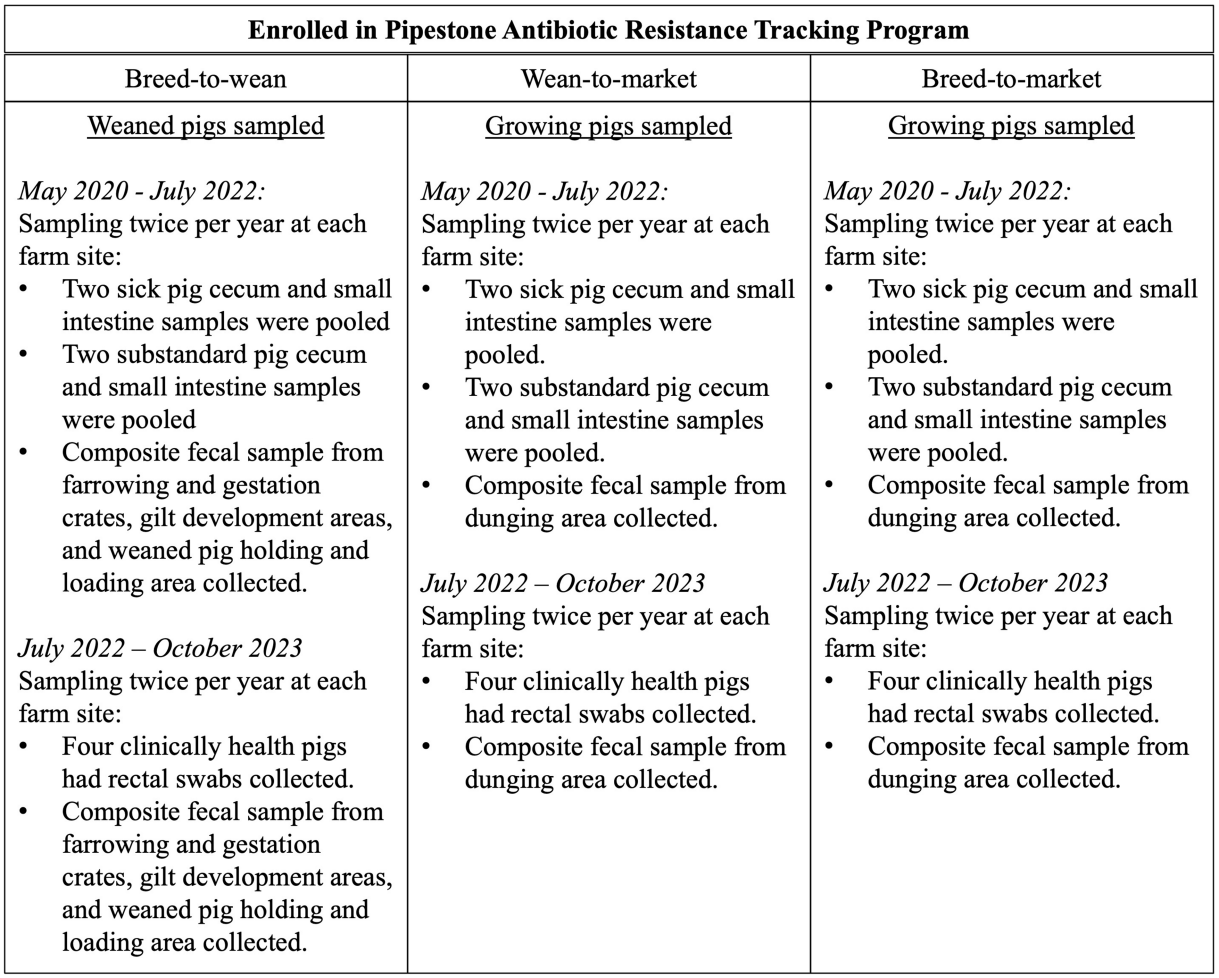
A description of the sampling plan for pig samples and composite fecal samples by farm type and time period.

Due to public health considerations, any meropenem- or colistin-resistant isolates identified through initial antibiotic susceptibility testing—based on minimum inhibitory concentrations and NARMS cut-off values—from composite fecal samples were subjected to sequencing. They were cultured in Tryptic Soy Broth (BD, Franklin Lakes, New Jersey) in preparation for DNA extraction using the DNeasy Blood & Tissue Kit (Qiagen, Hilden, Germany) for further analysis of these potentially concerning isolates. The quality and quantity of resulting DNA extracts were assessed via Qubit fluorometric analysis (Thermo Fisher Scientific, Waltham, Massachusetts). Using the manufacturer’s instructions, libraries were created using the DNA Prep kit and Nextera DNA CD indexes (Illumina, San Diego, California). Normalized libraries were then loaded onto the MiSeq platform (Illumina, San Diego, California) using MiSeq V3 600-cycle reagent kits (Illumina, San Diego, California). Sequence data were assembled using Bactopia v3.0.0 with default assembly settings (26). Assemblies were subjected to annotation for antimicrobial resistance genes using AMRFinderPlus with default settings, as implemented in Bactopia v3.0.0 (27). Outputs of AMRFinderPlus were analyzed for the presence of antimicrobial resistance genes annotated within the Pathogen Detection Reference Gene Hierarchy at the subclass level as either “COLISTIN” or “CARBAPENEM.” No such antimicrobial resistance genes were identified in any of the sequenced isolates. As a result, these isolates were retested by the US Department of Agriculture’s National Veterinary Services Laboratory (USDA NVSL) (Ames, Iowa) using the CMV5AGNF Gram-negative plate. The NVSL used a different lot of plates from the manufacturer than those used by the ADRDL. The NVSL results are used in this paper. The antibiotics and ranges tested are presented in Table 1. Results for *E. coli* and *Salmonella enterica* isolated from the sponge, intestinal samples, and fecal swabs are summarized in this paper (Figure 1).

**Table 1:** Minimum inhibitory concentrations (μg/ml) ranges for different antibiotics tested via various antimicrobial susceptibility testing plates produced by Thermo Fisher Scientific and used in the on-farm pig monitoring program.

	Bovine-porcine 7F plate	Gram-negative NARMS CMV3AGNF plate	Gram-negative NARMS CMV5AGNF plate	Used in pig medicine?
Amoxicillin/Clavulanic acid	Not tested	1/0.5 to 32/16	1/0.5 to 32/16	Yes
Ampicillin	0.25 to 16	1 to 32	1 to 32	Yes
Azithromycin	Not tested	0.12 to 16	0.25 to 64	No
Ceftiofur	0.25 to 8	0.12 to 8	Not tested	Yes
Cefoxitin	Not tested	0.5 to 32	1 to 32	No
Ceftriaxone	Not tested	0.25 to 64	0.25 to 64	No
Chloramphenicol	Not tested	2 to 32	2 to 32	No
Ciprofloxacin	Not tested	0.015 to 4	0.015 to 4	No
Clindamycin	0.25 to 16	Not tested	Not tested	Lincomycin
Colistin	Not tested	Not tested	0.25 to 8	No
Danofloxacin	0.12 to 1	Not tested	Not tested	No
Enrofloxacin	0.12 to 2	Not tested	Not tested	Yes
Florfenicol	0.25 to 8	Not tested	Not tested	Yes
Gamithromycin	1 to 8	Not tested	Not tested	No
Gentamicin	1 to 16	0.25 to 16	0.25 to 16	Yes
Meropenem	Not tested	Not tested	0.06 to 4	No
Nalidixic acid	Not tested	0.5 to 32	0.5 to 32	No
Neomycin	4 to 32	Not tested	Not tested	Yes
Penicillin	0.12 to 8	Not tested	Not tested	Yes
Spectinomycin	8 to 64	Not tested	Not tested	Yes
Streptomycin	Not tested	2 to 64	Not tested	No
Sulfisoxazole	Not tested	16 to 256	16 to 256	Other sulfonamide drugs
Sulfadimethoxine	256	Not tested	Not tested	Yes
Tetracycline	0.5 to 8	4 to 32	4 to 32	Yes
Tiamulin	0.5 to 32	Not tested	Not tested	Yes
Tilmicosin	2 to 16	Not tested	Not tested	Yes
Tildipirosin	1 to 16	Not tested	Not tested	No
Trimethoprim/ Sulfamethoxazole	2/38	0.12/2.38 to 4/76	0.12/2.38 to 4/76	Y (and other potentiated sulfonamides)
Tulathromycin	8 to 64	Not tested	Not tested	Yes
Tylosin tartrate	0.5 to 32	Not tested	Not tested	Yes
Total # of antibiotics tested	19	14	14	

### 2.5 Data management and analysis

A JavaScript application developed by Norsoft (Mankato, Minnesota) in collaboration with SDSU ADRDL integrated laboratory data into a structured query language (SQL) database hosted on a Pipestone Veterinary Services (PVS) server. An additional SQL statement was then executed from JMP 16.2 (SAS, Cary, North Carolina, USA) to extract data specific to this monitoring project, and additional attributes were added: NARMS consensus breakpoints as reported by the FDA in 2021^2^, FDA and World Health Organization (WHO) drug classifications, farm type, farm information, and farm identification codes used by Pipestone in PART. Then, the data were downloaded into a Microsoft Excel file (Microsoft, Redmond, Washington, USA). All results were compared to the laboratory report to ensure the file contained complete information and to identify any missing data or other issues. Any data issues were resolved with the ADRDL staff and corrected in the Excel file.

Descriptive statistics were calculated to summarize the number of farm systems, as well as their pig production and sample submission data. These were stratified across farm type and pig type to present the data without confounding those variables. AST data were reported as antibiotic-specific MIC values measured for each *E. coli* and *Salmonella* isolate collected from the composite fecal sponge swabs, pig intestinal samples, and fecal swabs. Isolate counts and percentages for each antibiotic and MIC value were determined and tabulated graphically as ‘squashtograms’ (see Supplementary files), which provide frequency and percentage data for isolates across different MIC values for each antibiotic tested. A handful of isolates were evaluated on Gram-positive NARMS plates rather than BOPO7F or NARMS Gram-negative plates. The antibiotics that overlapped were reported, and the rest of the data were excluded. All BTM farms were reclassified as WTM sites, as they were the only sites sampled at these enterprises. A total of 16 individual squashtograms were created—one for each combination of bacterial type (*E. coli* versus *S. enterica*), sample type (intestinal, fecal swab, or composite fecal sample), farm type (BTW vs. WTM), data period(Period 1 vs. Period 2), and pig type (substandard vs. sick). Human interpretative resistance breakpoints were indicated on the pig sample squashtograms, as no pig-specific breakpoints exist for *Enterobacterales* species from intestinal sources (28). For composite environmental fecal samples, the NARMS consensus breakpoints (also human-based) were used (see footnote 2), which are derived from the Clinical and Laboratory Standards Institute (CLSI) Performance Standards for Antimicrobial Susceptibility Testing (M100) (28). This allowed for the classification of the isolate as either resistant or susceptible (binary data). For the composite fecal samples, the frequency and percentage of isolates resistant based on breakpoints were also determined and stratified across farm types (BTW and WTM). Isolates with intermediate resistance were reclassified as susceptible in these calculations. A two-sample test of proportions, controlling for clustering at the farm enterprise level, was used to compare the percentage of resistance between farm types with a significance level of 0.05. The intra-class correlation coefficient (ICC) for the test was set to 0.28 based on the median of ICCs calculated for MIC values of *E.coli* and *Salmonella enterica* collected from swine farms in Alberta, Canada, in 2008 (29), as it was the best ICC measure available in the literature. Data manipulation and analysis were performed using Microsoft Excel version 16.75.2 (Redmond, Washington, USA) and both STATA 16.1 IC and STATA 18 BE (StataCorp, College Station, Texas, USA).

Data on antibiotic purchases were available through the PART program^3^ and were downloaded for the farms involved in AMR monitoring from May 2020 through October 2023. The data were then stratified and summarized by farm type (BTM, BTW, and WTM) and drug class. BTM site antibiotic purchase data were summarized separately, as antibiotics were purchased for both the BTW sites and WTM sites, making it impossible to differentiate which antibiotics were allocated to WTM or BTW sites. The total active ingredients of antibiotics purchased in milligrams (mg) were divided by the live weight in kilograms (kg) of pigs produced by that farm. This metric has been previously used to describe antimicrobial use in pig farms in the Midwest (30). For BTW Farms, the denominator was the product of the number of weaned pigs produced and their approximate weight (5.44 kg per pig) at the time of sale to WTM sites. For BTM and WTM farms, the denominator was the product of market pigs produced by their approximate weight (127.01 kg per pig) at the time of sale for harvest. The median, interquartile range, and range were calculated, and data were manipulated using JMP 16.2 (SAS, Cary, North Carolina, USA). Data was summarized from May 2020 through May 2021, June 2021 through June 2022, and July 2022 through October 2023.

## 3 Results

### 3.1 Farm enrollment and sampling summary

Sampling periods were planned based on funding cycles; however, farm sites were sampled outside of the expected periods due to competing medical priorities among the veterinary staff, as well as manpower and supply shortages. Table 2 summarizes the sampling completed. Period 1 sampling occurred from May 2020 through May 2021; however, it is likely that some Period 2 sampling also began during this period, based on third and fourth submissions from sites. Period 2 sampling extended into July 2022. Fecal swabs were used during Period 3, and sampling began in June 2022 and continued until October 2023 due to limitations in manpower and backorders on supplies in the winter of 2022/2023 and the spring of 2023, respectively.

**Table 2:** A summary of farm enterprises enrolled in antimicrobial resistance monitoring and the number of submissions per period of monitoring, May 2020 through October 2023.

	Breed-to-market		Wean-to-market		Breed-to-wean		Total
	#	%	#	%	#	%	# (%)
Number of producers							
Period 1	8	5.2	84	54.9	61	39.9	153 (100)
Period 2	8	5.3	83	54.6	61	40.1	152 (100)
Period 3	7	4.5	81	52.3	67	43.2	155 (100)
Number of cases							
Period 1	20	5.6	185	51.8	152	42.6	357 (100)
Period 2	15	5.8	148	57.4	95	36.8	258 (100)
Period 3	15	4.8	161	51.9	134	43.2	310 (100)

Number of pigs sold/producer	Median	Range	Median	Range	Median	Range
Period 1	54,232	16,250, 325,000	26,468	3,600, 327,958	100,466	10,431, 297,063
Period 2	54,232	16,250, 325,000	24,848	6,094, 354,717	111,117	18,252, 299,359
Period 3	86,827	36,000, 400,000	32,767	7,500, 442,650	158,253	28,164, 381,697

	# farms (%)	# submissions (%)	# farms (%)	# submissions (%)	# farms (%)	# submissions (%)
Period 1 submissions						
1	1 (12.5)	1 (5.0)	12 (14.3)	12 (6.5)	6 (9.8)	6 (3.9)
2	5 (62.5)	10 (50.0)	41 (48.8)	82 (44.3)	24 (39.3)	48 (31.6)
3	1 (12.5)	3 (15.0)	28 (33.3)	84 (45.4)	26 (42.6)	78 (51.3)
4	0 (0.0)	0 (0.0)	3 (3.6)	12 (6.5)	5 (8.2)	20 (13.2)
5	0 (0.0)	0 (0.0)	0 (0.0)	0 (0.0)	0 (0.0)	0 (0.0)
6	1 (12.5)	6 (30.0)	0 (0.0)	0 (0.0)	0 (0.0)	0 (0.0)
Total	8	20	84	190	61	152
Period 2 submissions						
1	2 (25.0)	2 (13.3)	28 (33.7)	28 (18.9)	32 (52.5)	32 (33.7)
2	5 (62.5)	10 (66.7)	46 (55.4)	92 (62.2)	24 (39.3)	48 (50.5)
3	1 (12.5)	3 (20.0)	7 (8.4)	21 (14.2)	5 (8.2)	15 (15.8)
4	0 (0.0)	0 (0.0)	2 (2.4)	8 (5.4)	0 (0.0)	0 (0.0)
5	0 (0.0)	0 (0.0)	0 (0.0)	0 (0.0)	0 (0.0)	0 (0.0)
6	0 (0.0)	0 (0.0)	0 (0.0)	0 (0.0)	0 (0.0)	0 (0.0)
Total	8	15	83	149	61	95
Period 3 submissions						
1	0 (0.0)	0 (0.0)	9 (11.1)	9 (5.6)	6 (9.0)	6 (4.5)
2	6 (85.7)	12 (80.0)	67 (82.7)	134 (83.2)	56 (83.6)	112 (83.6)
3	1 (14.3)	3 (20.0)	4 (3.9)	12 (7.5)	4 (6.0)	12 (9.0)
4	0 (0.0)	0 (0.0)	0 (0.0)	0 (0.0)	1 (1.5)	4 (3.0)
5	0 (0.0)	0 (0.0)	0 (0.0)	0 (0.0)	0 (0.0)	0 (0.0)
6	0 (0.0)	0 (0.0)	0 (0.0)	0 (0.0)	0 (0.0)	0 (0.0)
7	0 (0.0)	0 (0.0)	0 (0.0)	0 (0.0)	0 (0.0)	0 (0.0)
8	0 (0.0)	0 (0.0)	1 (1.2)	8 (5.0)	0 (0.0)	0 (0.0)
Total	7	15	81	163	61	134

In Period 1, 153 enterprises participated (8 BTM, 61 BTW, and 84 WTM), 152 enterprises participated (8 BTM, 61 BTW, and 83 WTM) in Period 2, and 155 enterprises participated (7 BTM, 67 BTW, and 81 WTM) in Period 3. The BTW sites produced more weaned pigs per site than the BTM and WTM enterprises produced market pigs, which was expected, as BTW sites often produce pigs for multiple WTM enterprises. The BTM enterprise sites produced more market pigs per site than the WTM enterprises alone. This production outcome occurred across all 3 years. The total number of submissions in Period 1 exceeded two submissions per farm, with a total of 20 submissions for the 8 BTM farms (2.5 per farm), 185 submissions for the 84 WTM farms (2.2 per farm), and 152 submissions for the 61 BTW farms (2.5 per farm). In Period 2, the total number of submissions was fewer than two, with 15 submissions from the 8 BTM farms (1.9 per farm), 148 submissions from the 83 WTM farms (1.8 per farm), and 95 submissions from the 61 BTW farms (1.6 per farm). In Period 3, each farm type contributed approximately two cases per site (BTM: 2.1, WTM: 2.0, and BTW: 2.0), as was originally planned (see Table 2).

### 3.2 Antibiotic purchases as a proxy for use

Tables 3–5 summarize purchased antibiotics by farm type and drug class for each period. The most commonly purchased antibiotics were those in the tetracycline class for all farm types across all periods. For BTM sites, the median milligrams purchased per kilogram of pig produced (mg/kg) ranged from 6.61 to 15.07 mg/kg. For WTM sites, it was 37.17 to 45.81 mg/kg, and for BTW sites, it was 36.48 to 59.81 mg/kg. For BTM sites, antibiotics in the penicillin class were the second most frequently purchased in Period 1 (median: 2.60 mg/kg) and Period 2 (median: 7.03 mg/kg), while antibiotics in the sulfonamide class were the most frequently purchased in Period 3 (median: 4.73 mg/kg). For the WTM enterprises, antibiotics in the penicillin class were the second most purchased antibiotic in all periods, with medians of 8.90, 12.04, and 9.69 mg/kg, respectively. For BTW facilities, penicillin antibiotics were the second most purchased in the first period (median: 12.29 mg/kg) and third period (median: 20.22 mg/kg), while antibiotics in the 16-member ring macrolide class were the second most purchased in the second period (median: 31.55 mg/kg). All farm types differed in the third most purchased class of antibiotic in the different time periods. For BTM, the third most commonly purchased antibiotics belonged to the pleuromutilin (tiamulin: administered via feed and water) or lincosamide (lincomycin: administered via injection, water, and feed) classes. For WTM, they were members of the sulfonamide or lincosamide classes. Finally, for BTW sites, the third most purchased antibiotics were antibiotics in the 16-member ring macrolide class, the lincosamide class, or the penicillin class. Tetracycline-class antibiotics used on pig farms included tetracycline (injectable), chlortetracycline (water and feed administration), and oxytetracycline (injectable and water administration). Penicillin antibiotics used included penicillin (injectable), ampicillin (injectable), and amoxicillin (water administration). Sulfonamide antibiotics (feed and water administration) include sulfadiazine and sulfamethoxazole. This drug class was also administered in combination with trimethoprim, a dihydrofolate reductase, as potentiated sulfonamides delivered via intravenous administration. In addition, sulfadimethoxine and sulfamethazine were also used alone as water medications. The 16-member ring macrolides used included tilmicosin (administered via feed and water), tylosin tartrate (administered via injectable feed and water), and tylavosin (administered via feed and water).

**Table 3:** Antibiotic purchases described by farm type and drug class in milligrams of drug used per kilogram of pig produced from May 2020 through May 2021.

Drug class	# farms with data / total farms (%)	Median mg/kg	Min mg/kg	25th Percentile mg/kg	75th Percentile mg/kg	Max mg/kg
Breed-to-market						
Aminoglycoside	7/8 (87.5)	0.37	0.06	0.14	1.04	3.44
Penicillin	8/8 (100)	2.60	0.13	0.42	6.68	21.65
Cephalosporin	7/8 (87.5)	0.16	0.07	0.14	0.39	0.42
DHFR Inhibitor	2/8 (25.0)	0.28	0.16	0.16	0.40	0.40
Fluoroquinolone	8/8 (100)	0.31	0.02	0.09	0.49	0.88
Lincosamide	7/8 (87.5)	1.46	0.12	0.88	5.80	27.62
Macrolide 15-member ring	5/8 (62.5)	0.02	0.00	0.01	0.12	0.21
Macrolide 16-member ring	5/8 (62.5)	0.60	0.04	0.25	1.98	3.24
Pleuromutilin	5/8 (62.5)	1.33	0.30	0.37	4.04	4.38
Sulfonamide	2/8 (25.0)	1.39	0.80	0.80	1.99	1.99
Tetracycline	7/8 (87.5)	6.61	1.00	1.00	26.80	31.21
Wean-to-market						
Aminoglycoside	74/84 (88.1)	1.92	0.05	0.59	4.61	15.06
Amphenicol	4/84 (4.8)	0.06	0.03	0.04	0.07	0.07
Penicillin	77/84 (91.7)	8.90	0.63	4.58	15.78	60.84
Cephalosporin	55/84 (65.5)	0.03	0.00	0.01	0.06	0.21
DHFR Inhibitor	66/84 (78.6)	0.62	0.03	0.36	1.28	3.60
Fluoroquinolone	76/84 (90.5)	0.20	0.02	0.10	0.38	1.28
Lincosamide	71/84 (84.5)	4.85	0.06	2.08	13.58	50.64
Macrolide 15-member ring	23/84 (27.4)	0.00	0.00	0.00	0.02	0.05
Macrolide 16-member ring	54/84 (64.3)	1.71	0.00^*^	0.75	5.72	51.55
Pleuromutilin	70/84 (83.3)	4.11	0.29	2.54	6.63	22.91
Sulfonamide	69/84 (82.1)	3.92	0.15	2.19	7.46	26.27
Tetracycline	77/84 (91.7)	37.17	1.64	12.48	71.22	326.89
Breed-to-wean						
Aminoglycoside	57/61 (93.4)	1.47	0.01	0.53	4.12	30.91
Amphenicol	1/61 (1.6)	4.23	4.23	4.23	4.23	4.23
Penicillin	58/61 (95.1)	12.29	2.08	6.60	18.14	51.94
Cephalosporin	51/61 (83.6)	0.72	0.01	0.23	3.06	21.31
DHFR Inhibitor	17/61 (27.9)	0.46	0.00	0.21	0.79	1.56
Fluoroquinolone	55/61 (90.2)	0.79	0.02	0.31	2.20	4.49
Lincosamide	57/61 (93.4)	9.51	0.78	4.85	16.74	66.83
Macrolide 15-member rings	30/61 (49.2)	0.47	0.00	0.11	1.01	8.59
Macrolide 16-member rings	50/61 (82.0)	5.61	0.11	1.04	28.26	1308.72
Pleuromutilin	33/61 (54.1)	6.34	0.00	2.00	24.06	78.94
Sulfonamide	18/61 (29.5)	3.00	0.00	1.62	5.42	47.54
Tetracycline	48/61 (78.7)	36.48	0.00^*^	5.12	323.97	754.54

DHFR is an abbreviation for dihydrofolate reductase and includes trimethoprim as a part of potentiated sulfonamide drugs. Amphenicols were not used on BTM. Macrolide 15-member ring antibiotics purchased include tulathromycin, and 16-member rings include tylosin, tilmicosin, and tylvalosin.

* Indicates cells with a minimum less than zero due to the return of purchased antibiotics, which then credited amounts back to farm accounts as a deduction.

**Table 4:** Antibiotic purchases described by farm type and drug class in milligrams of drug used per kilogram of pig produced from June 2021 through June 2022.

Drug class	# farms with data / total farms (%)	Median mg/kg	Min mg/kg	25th Percentile mg/kg	75th Percentile mg/kg	Max mg/kg
Breed-to-market						
Aminoglycoside	7/8 (87.5)	0.42	0.02	0.16	1.88	4.09
Penicillin	7/8 (87.5)	7.03	0.14	0.58	18.36	24.68
Cephalosporin	7/8 (87.5)	0.23	0.05	0.14	0.27	0.31
DHFR Inhibitor	6/8 (75.0)	0.36	0.00	0.03	0.58	0.88
Fluoroquinolone	7/8 (87.5)	0.34	0.02	0.23	0.39	1.04
Lincosamide	6/8 (75.0)	1.10	0.07	0.15	3.30	6.03
Macrolide 15-member ring	5/8 (62.5)	0.01	0.01	0.01	0.03	0.04
Macrolide 16-member ring	6/8 (75.0)	1.31	0.20	0.67	5.86	15.72
Pleuromutilin	7/8 (87.5)	2.26	0.33	0.55	2.63	12.69
Sulfonamide	6/8 (75.0)	1.77	0.01	0.13	2.87	4.36
Tetracycline	7/8 (87.5)	15.07	1.10	2.51	21.92	37.11
Wean-to-market						
Aminoglycoside	73/83 (88.0)	1.99	0.04	0.73	4.12	64.90
Amphenicol	8/83 (9.6)	0.05	0.01	0.02	0.08	0.37
Penicillin	78/83 (94.0)	12.04	0.07	7.76	21.22	120.86
Cephalosporin	63/83 (75.9)	0.04	0.00	0.02	0.07	0.29
DHFR Inhibitor	75/83 (90.4)	0.88	0.07	0.41	1.45	3.68
Fluoroquinolone	78/83 (94.0)	0.31	0.00	0.15	0.60	1.82
Lincosamide	73/83 (88.0)	4.87	0.05	1.94	11.37	59.76
Macrolide 15-member rings	24/83 (28.9)	0.01	0.00	0.00	0.02	0.07
Macrolide 16-member rings	64/83 (77.1)	4.87	0.00	2.20	9.16	35.37
Pleuromutilin	73/83 (88.0)	4.56	0.35	2.96	8.94	39.26
Sulfonamide	76/83 (91.6)	5.43	0.35	2.47	10.08	70.51
Tetracycline	74/83 (89.2)	45.81	1.35	19.38	85.57	338.10
Breed-to-wean						
Aminoglycoside	57/61 (93.4)	1.51	0.03	0.41	4.00	1123.09
Penicillin	60/61 (98.4)	15.07	0.00^*^	10.14	23.11	89.94
Cephalosporin	51/61 (83.6)	1.34	0.01	0.44	4.10	9.43
DHFR Inhibitor	26/61 (42.6)	0.83	0.09	0.49	1.80	6.10
Fluoroquinolone	52/61 (85.2)	1.07	0.00	0.42	3.36	11.20
Lincosamide	58/61 (95.1)	13.72	0.56	6.77	24.41	189.93
Macrolide 15-member rings	29/61 (47.5)	0.47	0.00^*^	0.09	1.54	4.03
Macrolide 16-member rings	55/61 (90.2)	31.55	0.18	6.85	152.85	812.24
Pleuromutilin	47/61 (77.0)	6.05	0.56	1.81	22.31	162.39
Sulfonamide	27/61 (44.3)	4.34	0.43	2.64	8.88	30.29
Tetracycline	51/61 (83.6)	59.81	0.33	18.60	230.88	1916.74

DHFR is an abbreviation for dihydrofolate reductase and includes trimethoprim as a part of potentiated sulfonamide drugs. Amphenicols were not used on BTM or BTW sites. Macrolide 15-member ring antibiotics purchased include tulathromycin, and 16-member rings include tylosin, tilmicosin, and tylavosin.

* Indicates cells with a minimum less than zero due to the return of purchased antibiotics, which then credited amounts back to farm accounts as a deduction.

**Table 5:** Antibiotic purchases described by farm type and drug class in milligrams of drug used per kilogram of pig produced from July 2022 through July 2023.

Drug class	# Farms with data / total farms (%)	Median mg/kg	Min mg/kg	25th Percentile mg/kg	75th Percentile mg/kg	Max mg/kg
Breed-to-market						
Aminoglycoside	6/7 (85.7)	0.42	0.16	0.28	1.21	1.61
Amphenicol	1/7 (14.3)	0.00	0.00	0.00	0.00	0.00
Penicillin	6/7 (85.7)	4.02	1.31	1.56	12.11	14.64
Cephalosporin	6/7 (85.7)	0.23	0.13	0.16	0.29	0.38
DHFR Inhibitor	3/7 (42.9)	0.53	0.19	0.19	1.38	1.38
Fluoroquinolone	6/7 (85.7)	0.59	0.07	0.26	0.83	1.03
Lincosamide	6/7 (85.7)	1.75	0.83	1.08	3.60	3.93
Macrolide 15-member ring	5/7 (71.4)	0.01	0.00	0.01	0.09	0.14
Macrolide 16-member ring	6/7 (85.7)	2.55	0.56	0.77	8.13	19.62
Pleuromutilin	5/7 (71.4)	1.29	0.19	0.48	8.99	10.15
Sulfonamide	4/7 (57.1)	4.73	0.97	1.38	10.89	12.24
Tetracycline	6/7 (85.7)	10.16	0.44	4.89	24.41	27.27
Wean-to-market						
Aminoglycoside	78/81 (96.3)	1.82	0.05	0.57	3.69	28.66
Amphenicol	8/81 (9.9)	0.06	0.00	0.01	0.17	0.18
Penicillin	76/81 (93.8)	9.69	0.11	5.45	17.77	52.20
Cephalosporin	67/81 (82.7)	0.03	0.00	0.01	0.07	0.16
DHFR Inhibitor	70/81 (86.4)	0.85	0.00	0.33	1.42	2.77
Fluoroquinolone	77/81 (95.1)	0.27	0.01	0.16	0.56	3.51
Lincosamide	72/81 (88.9)	4.22	0.11	1.39	12.12	62.49
Macrolide 15-member ring	30/81 (37.0)	0.01	0.00	0.00	0.03	0.22
Macrolide 16-member ring	58/81 (71.6)	4.61	0.02	1.89	7.91	103.33
Pleuromutilin	78/81 (96.3)	3.82	0.35	1.88	7.33	30.76
Sulfonamide	71/81 (87.7)	6.12	0.02	2.22	8.30	137.84
Tetracycline	76/81 (93.8)	43.55	3.56	23.07	71.30	265.21
Breed-to-wean						
Aminoglycoside	65/67 (97.0)	1.96	0.00	0.73	3.60	179.17
Penicillin	66/67 (98.5)	20.22	1.15	14.32	28.42	77.87
Cephalosporin	62/67 (92.5)	2.70	0.01	1.59	4.09	9.00
DHFR Inhibitor	40/67 (59.7)	1.26	0.10	0.55	2.79	6.31
Fluoroquinolone	57/67 (85.1)	1.01	0.01	0.31	3.70	8.85
Lincosamide	63/67 (94.0)	10.42	0.27	3.87	19.41	67.27
Macrolide 15-member ring	35/67 (52.2)	0.82	0.01	0.08	1.90	6.87
Macrolide 16-member ring	66/67 (98.5)	17.46	0.10	4.03	34.24	285.24
Pleuromutilin	56/67 (83.6)	7.51	1.19	2.95	12.12	111.05
Sulfonamide	41/67 (61.2)	6.45	0.50	2.47	14.02	36.70
Tetracycline	57/67 (85.1)	42.88	0.00	17.01	101.30	979.50

DHFR is an abbreviation for dihydrofolate reductase and includes trimethoprim as a part of potentiated sulfonamide drugs. Amphenicols were not used at either BTM or BTW sites. The macrolide antibiotics included tulathromycin (15-member ring) and tylosin, tilmicosin, and tylavosin (16-membered rings).

As for the percentage of farms that purchased an antibiotic rather than focusing on the amount of antibiotic purchased, florfenicol (amphenicol class) was the least commonly purchased, with 4.8, 9.6, and 9.9% of WTM sites purchasing in Periods 1, 2, and 3, respectively. Only one (1.6%) BTW site purchased florfenicol in Period 1. On the other hand, penicillin was one of the most commonly purchased antibiotics. Greater than 90% of BTW and WTM sites purchased penicillin in all periods. It was the most commonly purchased by BTW sites in all periods (tied with 16-member ring macrolides in period 3), by BTM sites in period 1, and tied with other antibiotic classes for most purchased in periods 2 and 3, and tied for the most commonly purchased on WTM sites in periods 1 and 2.

### 3.3 Antibiotic sensitivity testing

The squashtograms may be found in Supplementary Tables 1–16. Period 1 and 2 squashtograms are combined and stratified across *E. coli* and *S. enterica* isolates from intestinal samples, farm type (BTW, WTM), and sample source (sick pigs, substandard pigs). The Period 3 squashtograms are similar, but isolates are only from fecal swabs of healthy pigs. They stratify across farm types and bacteria (*E. coli* and *S. enterica*). The composite fecal samples were combined for all 3 years, as the sampling and testing methods remained unchanged, and these were then stratified by farm type.

For *E. coli* isolated from sick and substandard pig intestines tested on BOPO7F plates (Supplementary Tables 1–4; breakpoints from CSLI M100 indicated on table), WTM pig isolates for both sick and substandard pigs had > 10% more MIC values greater than or equal to the highest end of the antibiotic concentration range recorded for ampicillin, enrofloxacin, danofloxacin, florfenicol, gentamicin, neomycin, spectinomycin, sulfadimethoxine, tetracycline, and trimethoprim/sulfamethoxazole (TMS) compared to BTW isolates. The highest end of the tested range was at or above the CLSI M100 breakpoints for ampicillin, danofloxacin, enrofloxacin, florfenicol, gentamicin, sulfadimethoxine, tetracycline, and TMS. Sick pigs (*n* = 371) from WTM sites had > 10% more isolates with MIC values greater than or equal to the highest recorded levels, compared to substandard pigs (*n* = 365), for the fluoroquinolone antibiotics, enrofloxacin and danofloxacin. Almost all *E. coli* isolates from pig intestines had MIC values greater than or equal to the highest drug concentration tested for clindamycin, penicillin, tilmicosin, tiamulin, and tylosin tartrate, which is expected due to natural resistance (31). For BTW sites, most *E. coli* isolates from both sick (*n* = 247) and substandard (*n* = 248) pigs had MIC values at the lowest drug concentration tested for enrofloxacin and danofloxacin, gentamicin, neomycin, sulfadimethoxine, TMS, and tulathromycin. For WTM sites, most *E. coli* isolates from both sick and substandard pigs had MIC values at the lowest drug concentration tested for neomycin and tulathromycin. Greater than 70% of *E. coli* isolates from BTW sites and > 90% of *E. coli* isolates from WTM sites had MIC values greater than the highest drug concentration tested for tetracycline.

MIC values from the BOPO 7F plate for *S. enterica* isolates from the intestines of substandard pigs and sick pig samples stratified across farm types are found in Supplementary Tables 5–8. Similar patterns were seen in the MIC values between farm sites, as was the case with *E. coli*. Among substandard pig sample isolates, there were > 10% more isolates from WTM pigs than from BTW pigs with MIC values greater than or equal to the highest drug concentration levels for ampicillin, ceftiofur, enrofloxacin, danofloxacin, florfenicol, gentamicin, neomycin, spectinomycin, sulfadimethoxine, tetracycline, TMS, and tulathromycin. Sick pig results were similar, except a > 10% difference was not seen for sulfadimethoxine but was seen for tildipirosin, despite *Salmonella* having resistance to some macrolides (31). On the BTW farm sites, > 10% more isolates had MICs greater than or equal to the highest MIC value for tetracycline in sick pigs (*n* = 18) compared to substandard pigs (*n* = 23). Additionally, more than 10% of isolates had MIC values at the lowest MIC for gentamicin in substandard pigs compared to sick pigs. On WTM sites, there were no MIC comparisons between sick (*n* = 139) and substandard (*n* = 123) pigs that exhibited a > 10% difference. As expected, due to natural resistance, all *Salmonella* isolates from pig intestines collected at WTM and BTW sites had MIC values greater than the highest tested drug concentrations for clindamycin, tilmicosin, and tylosin tartrate, as well as at or above the highest concentration for penicillin and tiamulin (31). Greater than 40 and 60% of isolates had an MIC value for tetracycline greater than the highest concentration tested for substandard and sick pigs on BTW sites, respectively, and > 70% for both pig types on WTM sites. Most *Salmonella* isolates from sick and substandard pigs at BTW and WTM were found at sites with concentrations equal to or lower than the lowest measured concentrations for danofloxacin, enrofloxacin, gentamicin, and TMS.

Supplementary Tables 9, 10 summarize the MIC results from BOPO 7F plates for *E. coli* isolated from fecal swabs of healthy pigs during period 3 sampled at BTW (*n* = 524) and WTM (*n* = 672) farm sites, respectively. WTM sites had >10% more isolates than BTW sites, with MIC values at the highest drug concentrations for ampicillin, enrofloxacin, danofloxacin, gentamicin, neomycin, spectinomycin, sulfadimethoxine, tetracycline, and TMS. As expected, all BTW *E. coli* isolates’ MIC values for clindamycin, tiamulin, and tilmicosin were found to be greater than or equal to the highest drug concentration tested. For WTM sites, all isolates had MIC values greater than or equal to the highest drug concentration tested for clindamycin and penicillin, against which there is natural resistance (31), but also for sulfadimethoxine, tilmicosin, and tylosin. Greater than 70% of isolates from BTW and > 90% of isolates from WTM sites had MIC values greater than the highest concentration tested for tetracycline. For BTW and WTM sites, enrofloxacin, danofloxacin, TMS, and gentamicin had the most isolates at concentrations less than or equal to the lowest measured concentration, respectively.

As for *Salmonella* isolates from fecal swabs of healthy pigs (Supplementary Tables 11, 12), > 10% more of WTM (*n* = 65) isolates had MIC values greater than or equal to the highest drug concentration than BTW (*n* = 32) isolates for ampicillin, ceftiofur, florfenicol, gentamicin, neomycin, spectinomycin, tetracycline, tildipirosin, and TMS. As expected, all BTW *Salmonella* isolates’ MIC values for clindamycin, penicillin, tiamulin, tilmicosin, and tylosin (31) were found to be at or greater than the highest drug concentration tested. For WTM sites, 100% (*n* = 65) of isolates had MIC values greater than or equal to the highest drug concentration tested for clindamycin, gamithromycin, penicillin, tiamulin, tilmicosin, and tylosin. In total, 21.9% (7/32) of isolates from BTW and 72.3% (47/65) from WTM sites had an MIC value greater than or equal to the highest drug concentration for tetracycline. For both site types, enrofloxacin, danofloxacin, TMS, and gentamicin had the most isolates at concentrations less than or equal to the lowest measured concentration, respectively.

Table 6 summarizes the proportion of *E. coli* and *Salmonella* isolates collected from composite environmental swabs across all three periods. No meropenem or colistin resistance was detected in either bacterium when isolates were retested at USDA NVSL based on genotypic results. *E. coli* had higher rates of resistance in composite fecal samples from the WTM sites (*n* = 531) than BTW sites (*n* = 374) for all other antibiotics except for amoxicillin with clavulanic acid and cefoxitin and all antibiotics except cefoxitin among *S. enterica* (WTM: *n* = 14; BTW: *n* = 126) isolates. The highest proportion of *E. coli* isolates resistant to an antibiotic were resistant to tetracycline. The BTW and WTM sites had tetracycline resistance of 71.7% (268/374) and 88.0% (468/532), respectively. Among the *Salmonella* isolates, the highest percentages of resistance to tetracycline were observed in the BTW and WTM isolates, at 26.2% (33/126) and 66.0% (93/141), respectively. Complete squashtograms for these findings are summarized in Supplementary Tables 13–16.

**Table 6:** Proportion of *Salmonella* and *Escherichia coli* isolates from composite environmental swabs collected on pig farms in the upper Mid-West of the United States resistant to antibiotics on the National Antimicrobial Resistance Monitoring Systems gram negative plates (CMV3AGNF and CMV5AGNF) and breakpoints.

Antibiotic	***E. coli***			***Salmonella***		
	**Breed-to-wean**	**Wean-to-market**	***p*-value**	**Breed-to-wean**	**Wean-to-market**	***p*-value**
	#/total (%)	#/total (%)		#/total (%)	#/total (%)	
Amoxicillin/Clavulanic acid	46/374 (12.3)	83/530 (15.6)	0.3494	4/126 (3.2)	23/141 (16.3)	0.0039
Ampicillin	123/374 (32.9)	344/530 (64.9)	< 0.0001	9/126 (7.1)	70/141 (49.6)	< 0.0001
Azithromycin	24/374 (6.4)	96/530 (18.1)	0.0008	1/126 (0.8)	14/141 (9.9)	0.0085
Cefoxitin	47/374 (12.6)	94/530 (17.7)	0.1653	5/126 (4.0)	20/141 (14.2)	0.02
Ceftiofur^*^	22/179 (12.3)	65/258 (25.2)	0.0089	4/59 (6.8)	17/83 (20.5)	0.0409
Ceftriaxone	64/374 (17.1)	138/530 (26.0)	0.0369	6/126 (4.8)	30/141 (21.3)	0.0013
Chloramphenicol	38/374 (10.2)	169/531 (31.8)	< 0.0001	6/126 (4.8)	34/141 (24.1)	0.0003
Ciprofloxacin	66/374 (17.6)	249/531 (46.9)	< 0.0001	8/126 (6.3)	27/141 (19.1)	0.0119
Colistin^*^	0/195 (0.0)	0/272 (0.0)	NA	0/67 (0.0)	0/58 (0.0)	NA
Gentamicin	36/374 (9.6)	235/530^*^ (44.3)	< 0.0001	4/126 (3.2)	52/141 (36.9)	< 0.0001
Meropenem^*^	0/195 (0.0)	0/272 (0.0)	NA	0/67 (0.0)	0/58 (0.0)	NA
Nalidixic acid	45/374 (12.0)	177/530 (33.4)	< 0.0001	4/126 (3.2)	17/141 (12.1)	0.0286
Streptomycin^*^	60/178 (33.5)	158/260^*^ (60.7)	< 0.0001	11/59 (18.6)	53/83 (63.9)	< 0.0001
Sulfisoxazole	91/374 (24.3)	336/530 (63.4)	< 0.0001	27/126 (21.4)	78/141 (55.3)	< 0.0001
Tetracycline	268/374 (71.7)	467/531 (87.9)	< 0.0001	33/126 (26.2)	93/141 (66.0)	< 0.0001
Trimethoprim/ Sulfamethoxazole	31/374 (8.3)	259/530 (48.9)	< 0.0001	7/126 (5.6)	43/141 (30.5)	< 0.0001

One *E. coli* isolate accidentally tested on a gram-positive plate was tested for gentamicin (MIC = ≤ 16 μg/ml), and streptomycin (≤ 512 μg/ml) was excluded as it could not be classified into a susceptible, intermediate, or resistant category. Results were included where the antibiotic range tested allowed interpretation.

* Meropenem and colistin were added to the CMV5AGNF plate in October 2021, while streptomycin and ceftiofur were removed, which accounts for the sample number differences.

## 4 Discussion

The purpose of this project was to assess the feasibility of and present results from an on-farm pig monitoring program that included AST results from *E. coli* and *S. enterica* isolated from pig intestines (Periods 1 and 2), fecal swab samples (Period 3), and pig dunging area fecal composite samples (all three periods). The analysis in this study relied on farm enterprise antibiotic purchase data to estimate antibiotic use. The aforementioned samples were collected approximately twice a year from BTM (WTM-sampled), BTW, and WTM sites. The monitoring program revealed higher MIC values in isolates from pigs and in resistance from composite fecal sample isolates collected on WTM enterprises compared to BTW for both *E. coli* and *Salmonella*. In addition, *E. coli* isolates from sick pigs on WTM farms exhibited higher MIC values than those from substandard pigs. Tetracycline antibiotics were the most frequently purchased antibiotics across all farm types, although a greater number of farms overall purchased antibiotics from the penicillin class. Comparison of drug purchases in terms of mg of antibiotic per kg of pig produced across farm types is also difficult, as BTW farms report only weaned pigs as output, while treatments are also administered to sows and gilts.

### 4.1 Sampling limitations

On-farm monitoring for AMU and AMR is challenging. Such programs require significant cost, time, and effort for sample collection, project administration, data management, and data analysis. Sample sizes for this program were driven by logistical and economic constraints and were not of a scale that would be representative. From a practical standpoint, the program described herein was resource-intensive, yet it did not necessarily provide a return on investment for pig producers, even though it provided useful information for AMR research and monitoring. The program did not differentiate between commensal and pathogenic strains of *E. coli* or provide representative information on AMR patterns that could guide clinical decisions. The system was not designed for such purposes, and, as a result, the monitoring system had limited returns to the veterinary team and the producers involved. Managing sample collection timeframes also presented a challenge. Samples were collected by veterinarians during their regularly scheduled farm visits, which often occurred outside the desired monitoring timeframe. For example, some samples designated for collection during Period 2 were likely collected at the end of Period 1, as a farm visit was needed at that time. Table 2 shows that 25.0% of BTW, 32.6% of WTM, and 52.5% of BTW sites submitted only one sample in Period 2, and this was because 12.5% of BTW, 33.3% of WTM, and 42.6% of BTW sites collected three samples during Period 1, with the third sample likely meant for Period 2. Future modifications to the program should allow for sampling by farm staff rather than relying solely on professional veterinarian visits to improve the timing of sampling.

In the monitoring system described, BTM (WTM sites sampled) and WTM enterprise sampling further limited interpretability for producers, as they often had a different site sampled at each visit. Each farm visit also included two pooled samples derived from four pigs or one pooled sample from four swabs. The pooling reduced statistical power, as only one representative colony derived from two to four samples was tested, and it potentially limited the capture of bacterial diversity at a site. This is further exacerbated by the fact that fecal swabs from healthy pigs and tissues of substandard pigs may not have had a dominant clone. That said, if a clear difference in morphology was observed among colonies on a plate, then multiple representative colonies were tested for AST.

From this system design, we could report what was detected but could not determine if the results were common, representative, or outliers. It is not possible to provide patterns over time, given that many sites were sampled once per year and that samples were pooled. More frequent sampling by barn staff from the same BTW barn and from WTM barns that receive pigs from that BTW source would improve the program. Testing of individual samples rather than pooled samples would allow for greater diversity capture, the ability to trend over time, an understanding of how and where resistance develops, and more representative summaries of the farms included in the study.

A limitation of our study design is that pig ages, treatments, and disease events were not controlled for or recorded in this monitoring system. Additionally, it is unknown if the samples in the WTM production stage over- or under-represent different ages of pigs. Younger pigs were likely sampled more often, as they are less expensive to sacrifice (substandard pigs) and tend to have more health problems when changing diets and adjusting to larger group housing during the transition from litter-based housing. Although there was no feasible method to sample pigs randomly, the use of purposive and convenience sampling could also introduce selection biases. Sampling within age groups using a random selection of pens could reduce those biases. A previous study (32) and a recently published scoping review (33) showed a decline in AMR prevalence as pigs and other livestock age. Piglets arrived in the WTM system at 21 to 28 days of age and were started on feed. There are significant changes and establishment of the pig gut microbiome within the first 2 to 3 weeks on feed (34). Controlling for age, treatment, and disease events, and incorporating a random component into sampling could enhance the monitoring program.

### 4.2 Antimicrobial resistance summary

There have been few other on-farm monitoring systems reported, and none with both AMR and AMU solely based on farm-level data. Canada has a voluntary on-farm monitoring system in place for pig farms across five provinces. Farms collect a single composite fecal sample from the oldest pens of pigs in the grow-finish sector annually. Veterinarians collect samples from different farms throughout the year to capture seasonal variations. Samples in that program were cultured for *E. coli*, *S. enterica*, and *Campylobacter* species, and all were evaluated on NARMS broth microdilution plates. NARMS breakpoints were used to determine resistance, which was primarily based on the CLSI M100 guidance. Ciprofloxacin breakpoints were from the European Union Committee on Antimicrobial Susceptibility Testing (35).

In the present study, the interpretation of MIC values obtained from AST testing of pig isolates of *E. coli* and *S. enterica* was limited, and this had an impact on the data interpretation of this monitoring system. There were no breakpoints for common pig pathogens from the intestine for these pathogens. Instead, human breakpoints from isolates obtained from urinary tract infections in humans were used to indicate potential breakpoints (28) on the tabulated and graphical squashtograms. However, no analysis concerning resistance to isolates from pig samples for veterinary purposes was provided.

There is intrinsic resistance of both *S. enterica* and *E. coli* to clindamycin and macrolides (tulathromycin, gamithromycin, tildipirosin, tilmicosin, and tylosin tartrate), except for azithromycin and *S. enterica*. Furthermore, *S. enterica* is generally susceptible to aminoglycosides (gentamicin, neomycin, and streptomycin), first- and second-generation cephalosporins, and cephamycins (cefoxitin) *in vitro*. However, they are not clinically effective (31). This explains why many isolates tested for susceptibility to clindamycin and the macrolides had MIC values at or greater than the highest concentration tested. Tiamulin, a pleuromutilin, also had MIC values reported mostly at the high end of the tested range. There is no known resistance to this antibiotic, and no breakpoint has been established. It is unclear if the range is inadequate or if the isolates have resistance.

Finally, with *E. coli* isolates from intestinal samples, there were more isolates above the recommended CLSI human breakpoint among sick pigs compared to substandard pigs on WTM farms. A study published in 2023 suggests that fluoroquinolone use is associated with higher MIC values in *E. coli* (36) when farm clustering was controlled for, as well as confounding by production stage (WTM and BTW) and quarter of the year. If the sick pigs were treated with fluoroquinolone antibiotics, this could provide a testable hypothesis as to why the percentage of resistance in sick pigs was higher. In a cattle study, the odds of recovering a fecal *E. coli* isolate resistant to ceftiofur after treatment were increased for 13 to 15 days post-exposure (37). This further highlights the need to track treatment information in monitored barns to control for any resistance-associated confounding. In this study, we also saw an increased resistance in composite-fecal and pig-origin *E. coli* and *Salmonella* species isolates from WTM sites compared to BTW sites. NARMS breakpoints were applied to the composite fecal sample isolates tested on the NARMS panel. This may be a suitable consideration for public health, but it is unclear how well those interpretations apply to pig medicine.

### 4.3 Antimicrobial purchased data summary

In the program described in this paper, the amount of drug administered per kilogram of animal was used as an aggregated endpoint. This was done as drug use is not necessarily consistent, and estimating the per-day usage might give that impression. Often, treatments are performed in defined periods. Canada also uses a daily defined dose, calculated as the mg of drug divided by the kilograms of pig produced per day, utilizing national-level herd data and antimicrobial sales collected by the government from importers, compounders, and manufacturers. AMU data were not collected at the farm level (35) in that program. Furthermore, there are multiple AMU monitoring programs in Europe where the metric for measurement has not been standardized, although many are based on mg/kg measurements (38).

Summary statistics for purchased antibiotics were done as reported by other previously (30); the total milligrams of purchased antibiotics were divided by the mass of pigs in kilograms produced at each production stage. The previous study also showed that tetracyclines were used much more commonly on WTM farms compared to other antibiotics. Beyond tetracyclines, there was considerable variability; however, similar to the results in the present study, other commonly used antibiotics included lincosamides, penicillins, pleuromutilins, and macrolide antibiotics.

Considering the results from this study, comparisons between site types should be avoided. On BTW sites, the AMU metric included weaned pigs in the denominator; however, the population of the site receiving antibiotics included sows, gilts (female pigs that have not farrowed), and vasectomized or castrated boars used to monitor heat cycles of sows and gilts. In contrast, the WTM metric calculated both antibiotic usage and pig production based solely on pigs grown for market. For BTM sites, antibiotics were purchased for both their BTW and WTM sites (and associated pig types) and summarized based on the kilograms of market pigs produced. One would expect the metric to report higher numbers of purchased antibiotics than WTM sites alone, but in this study, that did not occur. Another challenge occurs with this metric when sites experience a severe disease outbreak. These outbreaks led to the on-site treatment of all pigs with antibiotics, often administered through feed and water. These herds may also experience high abortion rates and mortality rates, leading to a reduction in the output of pigs produced. Consequently, the denominator decreases significantly while the numerator increases, resulting in very high mg/kg estimates. This is likely the cause of the very high maximum values observed in Table 3. For example, in some BTW sites during Period 2, up to 1916.74 mg of tetracycline and 812.24 mg of 16-membered ring macrolides were purchased per kilogram of weaned pig produced.

### 4.4 Recommendations for future programs

On-farm monitoring programs would ideally control for age, disease status, time since treatment, treatments received, geography, and farm size in their design to allow for a better comparison of AMR between similar-aged pigs while controlling for disease presence on the farm. This would enable a better understanding of results based on pig-level characteristics rather than just the production stage. Future research would benefit from tracking and controlling for disease events, as well as from more granular data on antimicrobial use.

This research supports other scientific findings that there are often difficult-to-interpret differences in AMR and AMU between production stages and emphasizes some of the challenges with on-farm monitoring, particularly when the return on investment to the farmer is not evident. These programs are critical to understanding AMR development, and the lessons learned from this work can be used to enhance future monitoring programs.

The lack of breakpoints contributed to challenges when interpreting AST results received from veterinary diagnostic laboratories and to the interpretability of the results. Some diagnostic laboratories report whether the MIC value of the isolate indicates if the pathogen is susceptible (S), has intermediate resistance (I), or is resistant (R), but they do not describe the source of the software used to define breakpoints. Additionally, while compiled susceptibility results for pig pathogens from veterinary diagnostic laboratories can be found online, there are often no descriptions of how breakpoints were defined, which limits reliability and interpretation. It is not recommended to present S-I-R data in compilation or for individual results without describing how the designations were determined for the recipient (39, 40). There is likely a misinterpretation of results from reports with S-I-R information that are not transparent in their methodology, particularly for bacteria-antibiotic data with no defined breakpoints in pig medicine and no description of how these breakpoints were determined. The lack of pig-specific breakpoints and non-transparent S-I-R designations limits veterinary antibiotic stewardship, clinical decision-making, and on-farm monitoring capabilities. This lack of standardization makes it challenging to assess AMR in veterinary pathogens and to make informed decisions regarding veterinary antimicrobial use. There are significant limitations to our ability to assess the MIC data presented here and to understand the status of bacteria isolated from the monitoring program.

Another interpretative option was to calculate epidemiological cut-off (ECOFF) values. This requires a wide enough range of antibiotic concentrations to be tested to meet ECOFF calculation assumptions and that multiple laboratories be used to conduct the testing. The BOPO 7F veterinary plates had limited ranges of MICs, and only one laboratory was used. The criteria for ECOFF calculations that were not met included isolates representing two MIC concentrations on either side of the mode, the modal MIC value cannot be the lowest or highest MIC, and at least three different diagnostic laboratories had to contribute (41, 42). A pig-specific broth microdilution plate with wider ranges may alleviate some of these problems if this is attempted in the future.

Potential improvements to these interpretative challenges would be the development of breakpoints for antibiotics used to treat pig pathogens in the most affected organ systems. Of course, for *E. coli,* these may be applied to commensal organisms. Additionally, the use of a porcine-specific MIC plate with a wider range of concentrations for key pathogens would improve the calculation of ECOFF values. Clinicians and researchers alike would benefit from the development of veterinary breakpoints and ECOFF values for a broader range of antimicrobials and bacterial species. There is an effort underway for the latter by the VetCAST, a subcommittee of the European Union’s Committee on Antimicrobial Susceptibility Testing (43), and by Iowa State University in Ames, Iowa (44). Finally, the most practical solution is to provide veterinarians with a better understanding of how to interpret AST results and the strengths and limitations of the laboratory data presented to them when making clinical decisions.

## Data Availability

MIC and antibiotic purchase data may be made available upon reasonable request all of which would be de-identified prior to sharing. Sequence data can be found at: https://www.ncbi.nlm.nih.gov/ using SRA numbers SRS22840164, SRS22840163, SRS22840162, SRS22840161, SRS22840160, SRS22840159, and SRS22840156.

## Footnotes

^1^https://www.aphis.usda.gov/aphis/ourfocus/animalhealth/monitoring-and-surveillance/nahms/antimicrobial-use-resistance, Accessed August 16, 2024.

^2^https://www.fda.gov/media/108180/download; Accessed June 25, 2023.

^3^https://www.pipestone.com/part/, Accessed February 1, 2024.
